# Clinical Outcomes Associated with GLP-1 Receptor Agonist Exposure in Non-Diabetic Patients with Chronic Pancreatitis: A Retrospective Cohort Study

**DOI:** 10.3390/jpm16080437

**Published:** 2026-08-20

**Authors:** Arkadeep Dhali, Jyotirmoy Biswas, Fayaz Khan, Dushyant Singh Dahiya, Saikat Mandal

**Affiliations:** 1 Academic Unit of Gastroenterology, Sheffield Teaching Hospitals NHS Foundation Trust, Sheffield S5 7AU, UK; 2 School of Medicine, Dentistry and Biomedical Sciences, Queen’s University of Belfast, Belfast BT9 7BL, UK; 3 Department of Medicine, Barasat Government Medical College and Hospital, Barasat 700124, India; 4 Department of Palliative Medicine, Roswell Park Comprehensive Cancer Center, Buffalo, NY 14263, USA; 5 Division of Gastroenterology, Hepatology & Motility, School of Medicine, The University of Kansas, Kansas City, KS 66160, USA; 6 School of Medicine, University of Nottingham, Nottingham NG7 2UH, UK

**Keywords:** chronic pancreatitis, cohort, GLP-1 analogue, pharmacoepidemiology, pancreatic safety

## Abstract

**Background**: GLP-1 receptor agonists (GLP-1 RAs) are increasingly used for obesity and metabolic disease, but their use in people with chronic pancreatitis is not a chronic pancreatitis-directed indication and direct evidence is limited. We described recorded outcomes among non-diabetic adults carrying a chronic pancreatitis diagnosis who did or did not have recorded GLP-1 RA exposure. **Methods**: We performed a retrospective propensity score-matched cohort study using the TriNetX Collaborative Network. Chronic pancreatitis was identified from a recorded diagnosis; supporting imaging, histological, functional, or specialist-confirmation criteria were unavailable. The exposed cohort included patients receiving dulaglutide, semaglutide, or tirzepatide (*n* = 1441 before matching), and the comparator cohort included patients without recorded GLP-1 RA exposure (*n* = 142,047 before matching). One-to-one propensity score matching generated 1422 patients in each cohort. Outcomes were assessed from 1 to 1095 days after the index date using risk comparisons and time-to-event analyses. **Results**: Mean follow-up after matching was 458.8 days in the exposed cohort and 664.4 days in the comparator cohort. Recurrent acute pancreatitis was recorded in 19/799 (2.4%) versus 76/704 (10.8%) patients (HR 0.247, 95% CI 0.149–0.409), pancreatic cancer in 10/1373 (0.7%) versus 40/1345 (3.0%) (HR 0.255, 95% CI 0.128–0.511), and all-cause mortality in 21/1419 (1.5%) versus 157/1416 (11.1%) (HR 0.167, 95% CI 0.105–0.263). A similar direction was observed across several coded outcomes. Vitamin D deficiency showed a higher hazard (HR 1.556, 95% CI 1.129–2.145), although its risk comparison was not significant. **Conclusions**: These estimates describe outcomes in a selected treatment-exposed phenotype of chronic pancreatitis. The findings are hypothesis-generating and should not guide prescribing decisions. Further prospective studies are required to confirm this hypothesis.

## 1. Introduction

Chronic pancreatitis (CP) is an uncommon but high-burden disease with substantial long-term morbidity and reduced survival in population-based studies. Pain phenotype, disease-related complications, disability, and healthcare utilization are major determinants of impaired quality of life in CP, while pancreatic exocrine insufficiency has independently been associated with excess mortality [1,2,3]. CP also confers an elevated long-term risk of pancreatic cancer, making identification of therapies that do not worsen pancreatic outcomes particularly important [4].

Incretin-based therapies have transformed the treatment of obesity and metabolic disease, with semaglutide and tirzepatide producing substantial and sustained weight loss in large randomized trials [5,6]. However, pancreatic safety has remained a clinical concern. Early signal-detection and population-based case–control studies raised concern regarding pancreatitis and other pancreatic adverse events with incretin-based therapies [7,8], whereas more recent population-based cohort data have not shown an excess pancreatic cancer incidence among GLP-1 receptor agonist users with type 2 diabetes [9]. These data arise predominantly from obesity or diabetes populations, so by inference patients with established CP, particularly those without diabetes, remain poorly studied. Against this background, we designed this study as an exploratory association analysis of recorded GLP-1 RA exposure, rather than as an evaluation of GLP-1 RAs as CP-directed therapy, in order to describe pancreatic, healthcare utilization, and other clinically relevant outcomes in non-diabetic adults with established CP.

## 2. Methods

### 2.1. Study Design and Data Source

We performed a retrospective, propensity score-matched cohort study using the TriNetX US Collaborative Network, a federated research platform containing de-identified electronic health records, including diagnoses, procedures, medications, laboratory values, and genomic information, from large healthcare organizations across the United States. The study search was performed in February 2026.

### 2.2. Study Population and Cohort Definition

Adults aged 18 years or older with a recorded diagnosis of chronic pancreatitis were identified. The cohort definition did not require repeated diagnostic coding, imaging, histological confirmation, pancreatic function testing, or specialist adjudication; therefore, the study population represents patients carrying a recorded chronic pancreatitis diagnosis rather than a clinically validated specialist cohort. The exposed cohort included patients with chronic pancreatitis who had at least one recorded exposure to dulaglutide, semaglutide, or tirzepatide and no recorded type 1 or type 2 diabetes mellitus. This cohort comprised 1441 patients before matching. The comparator cohort included patients with chronic pancreatitis, no recorded GLP-1 receptor agonist exposure, and no recorded type 1 or type 2 diabetes mellitus; it comprised 142,047 patients before matching. GLP-1 RA exposure was treated strictly as a recorded medication exposure. TriNetX did not provide the prescribing indication, and the analysis could not distinguish obesity treatment from other non-diabetes indications.

### 2.3. Index Event, Follow-Up, and Matching

**Figure 1:**
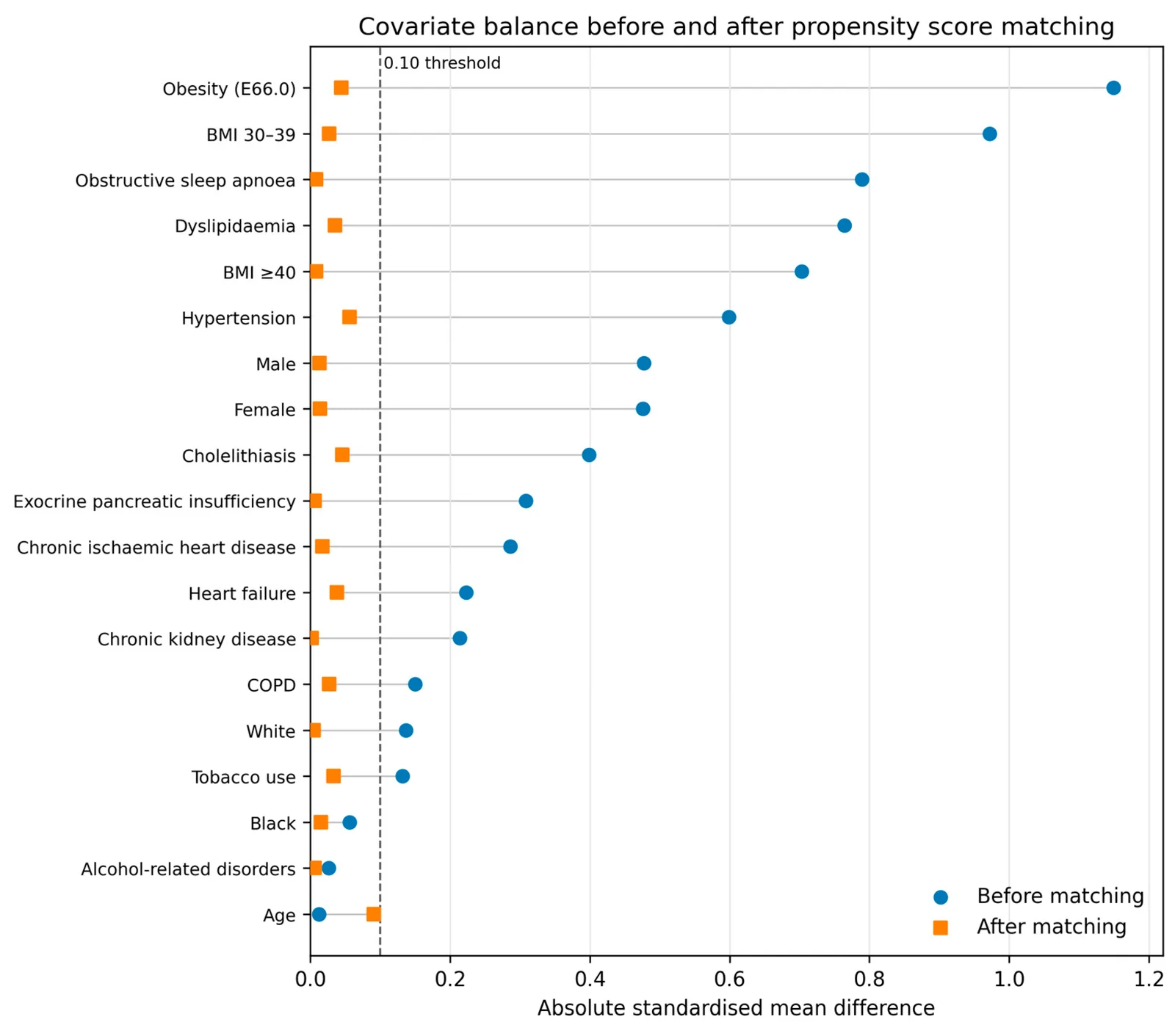
Love plot showing absolute standardized mean differences before and after propensity score matching. The dashed line indicates the prespecified balance threshold of 0.10.

The index event was the date on which each patient first met all cohort criteria. In the GLP-1 receptor agonist cohort, this corresponded to the date on which chronic pancreatitis and GLP-1 receptor agonist exposure criteria were simultaneously satisfied. In the non-GLP-1 receptor agonist cohort, the index event was the first chronic pancreatitis diagnosis in the absence of GLP-1 receptor agonist exposure and diabetes. Outcomes were assessed from 1 day to 1095 days after index. Index events were restricted to the preceding 20 years, although no patients were excluded on that basis. To reduce baseline imbalance, 1:1 nearest-neighbour propensity score matching using a greedy algorithm was performed within TriNetX. The propensity score incorporated demographics and baseline comorbidities, including hypertension, chronic kidney disease, chronic ischemic heart disease, heart failure, atrial fibrillation/flutter, chronic obstructive pulmonary disease, dyslipidemia, tobacco use, alcohol-related disorders, obesity-related diagnoses, obstructive sleep apnoea, cholelithiasis, pancreatic steatorrhoea, and exocrine pancreatic insufficiency. After matching, 1422 patients remained in each cohort (Table 1). Covariate balance was assessed using absolute standardized mean differences, with values below 0.1 considered adequately balanced; pre- and post-match values are displayed in a Love plot (Figure 1). Available weight-related variables and recorded tobacco use were included in matching, but weight trajectory, smoking intensity, and smoking-cessation efforts were not available. The asymmetric index definition is an important design limitation because patients in the exposed cohort had to survive and remain observable until GLP-1 RA exposure was recorded, whereas comparator patients entered follow-up at chronic pancreatitis diagnosis. Time-zero, immortal-time, and survivor biases therefore cannot be excluded.

### 2.4. Outcomes and Statistical Analysis

Outcomes were assessed in the post-index period using both risk analysis and Kaplan–Meier survival analysis. Patients with the outcome before the analysis window were excluded from that outcome-specific analysis so that only incident recorded events were captured. Outcomes included recurrent acute pancreatitis, pancreatic cancer, all-cause mortality, healthcare utilization outcomes (emergency room visits, hospitalization, and ERCP), opioid-related outcomes, gastrointestinal symptoms, hepatobiliary outcomes, metabolic and endocrine outcomes, nutritional and hematological outcomes, and cardiovascular and renal outcomes. Risk analysis generated risks, risk differences, risk ratios, and odds ratios with 95% confidence intervals. Kaplan–Meier analyses were censored at the last recorded fact in the medical record, and groups were compared with the log-rank test. Hazard ratios with 95% confidence intervals were derived from Cox proportional hazards models, and proportionality was assessed using Schoenfeld residuals. A two-sided *p*-value below 0.05 was considered statistically significant. As a post hoc sensitivity analysis for unmeasured confounding, E-values were calculated for the principal outcomes using the reported hazard ratios and 95% confidence intervals. For protective associations, hazard ratios and confidence interval limits were inverted before E-value calculation. E-values were interpreted as the minimum strength of association that an unmeasured confounder would need to have with both GLP-1 RA exposure and the outcome, conditional on measured covariates, to explain away the observed association [10]. Because the study used de-identified TriNetX data, it was exempt from institutional review board approval. No causal estimand was specified; the analyses describe associations between recorded exposure and recorded outcomes rather than treatment efficacy or safety. The database did not allow validation of prescribing indication, dose, duration, adherence, persistence, switching, time-varying exposure, surveillance intensity, or chronic pancreatitis severity. Risk and survival analyses use different estimands and censoring structures; therefore, discordant statistical significance between these approaches was not interpreted as evidence of a treatment effect.

## 3. Results

### 3.1. Baseline Characteristics

Before matching, the GLP-1 receptor agonist cohort and the non-GLP-1 receptor agonist cohort differed substantially. The exposed cohort had a higher proportion of women (72.7% vs. 50.1%; standardized mean difference [SMD], 0.476) and higher prevalences of obesity-related diagnoses, including obesity due to excess calories (45.0% vs. 2.6%; SMD, 1.149), body mass index 30–39 (40.5% vs. 4.1%; SMD, 0.972), body mass index ≥ 40 (22.9% vs. 1.3%; SMD, 0.703), obstructive sleep apnoea (31.9% vs. 3.8%; SMD, 0.789), and dyslipidemia (54.8% vs. 20.3%; SMD, 0.764). Mean age was similar (54.0 ± 13.3 vs. 54.2 ± 17.7 years; SMD, 0.012). The exposed cohort also had higher prevalences of hypertension (58.4% vs. 29.9%; SMD, 0.599), chronic ischemic heart disease (17.6% vs. 8.1%; SMD, 0.286), heart failure (10.3% vs. 4.5%; SMD, 0.223), chronic kidney disease (11.5% vs. 5.5%; SMD, 0.214), cholelithiasis (21.2% vs. 7.5%; SMD, 0.399), and exocrine pancreatic insufficiency (6.3% vs. 0.7%; SMD, 0.308). After matching, all available baseline covariates had SMDs below 0.1 (Figure 1). Available weight proxies were balanced after matching (SMD 0.044 for obesity, 0.027 for BMI 30–39, and 0.008 for BMI ≥ 40), as was recorded tobacco use (SMD 0.033). Age at the index event was 54.0 ± 13.3 versus 55.3 ± 15.4 years (SMD, 0.091), and 72.5% versus 73.1% were women. Mean follow-up after matching was 458.8 ± 348.5 days (median, 396; IQR, 587) in the GLP-1 receptor agonist cohort and 664.4 ± 422.8 days (median, 754; IQR, 864) in the comparator cohort, with a difference of 205.6 days (30.9% shorter relative to the comparator mean).

**Figure 2:**
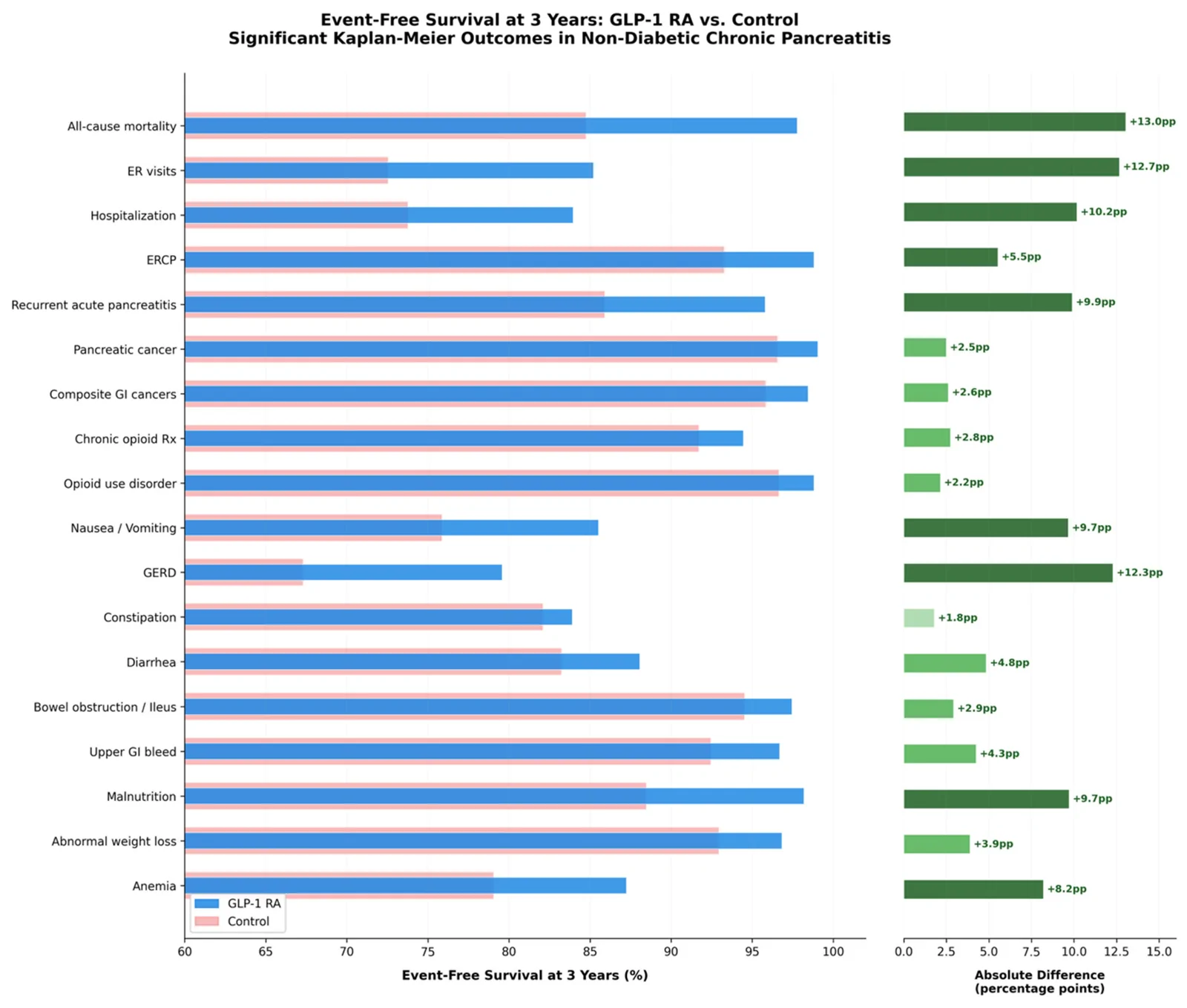
Event-free survival at three years.

The key outcomes are summarized in Table 2. Event-free outcomes at the end of three years are summarized in Figure 2.

### 3.2. Pancreatic, Oncologic, Mortality, and Healthcare Utilization Outcomes

After excluding patients with prior acute pancreatitis, recurrent acute pancreatitis was recorded in 19 of 799 patients (2.4%) in the GLP-1 receptor agonist cohort and 76 of 704 patients (10.8%) in the comparator cohort (risk ratio [RR], 0.220; 95% CI, 0.135–0.360; hazard ratio [HR], 0.247; 95% CI, 0.149–0.409; log-rank *p* < 0.001). Incident pancreatic cancer was recorded in 10 of 1373 patients (0.7%) versus 40 of 1345 patients (3.0%) (RR, 0.245; 95% CI, 0.123–0.488; HR, 0.255; 95% CI, 0.128–0.511; *p* < 0.001). All-cause mortality was recorded in 21/1419 patients (1.5%) versus 157/1416 patients (11.1%) (RR, 0.133; 95% CI, 0.085–0.209; HR, 0.167; 95% CI, 0.105–0.263; *p* < 0.001). Emergency room visits were recorded in 43/578 (7.4%) versus 121/614 (19.7%) patients (RR, 0.378; 95% CI, 0.272–0.525; HR, 0.441; 95% CI, 0.311–0.626; *p* < 0.001), hospitalization in 42/601 (7.0%) versus 103/528 (19.5%) (RR, 0.358; 95% CI, 0.255–0.503; HR, 0.428; 95% CI, 0.298–0.615; *p* < 0.001), and ERCP in 11/1298 (0.8%) versus 70/1272 (5.5%) (RR, 0.154; 95% CI, 0.082–0.289; HR, 0.166; 95% CI, 0.088–0.313; *p* < 0.001). In post hoc E-value analysis, the point-estimate E-values were 7.56 for recurrent acute pancreatitis, 7.31 for pancreatic cancer, and 11.45 for all-cause mortality. The corresponding E-values for the confidence interval limits closest to the null were 4.32, 3.33, and 7.07, respectively. The exposed-group event counts for recurrent acute pancreatitis (*n* = 19), pancreatic cancer (*n* = 10), and mortality (*n* = 21) were small and are reported to emphasize the exploratory nature of these estimates.

### 3.3. Opioid-Related and Gastrointestinal Outcomes

Chronic opioid prescriptions occurred in 30 of 1161 patients (2.6%) in the GLP-1 receptor agonist cohort and 69 of 1139 patients (6.1%) in the comparator cohort (RR, 0.427; 95% CI, 0.280–0.650; HR, 0.522; 95% CI, 0.338–0.804; *p* = 0.003). Opioid use disorder occurred in 10 of 1298 patients (0.8%) versus 30 of 1330 patients (2.3%) (RR, 0.342; 95% CI, 0.168–0.696; HR, 0.437; 95% CI, 0.212–0.898; *p* = 0.021). The GLP-1 receptor agonist cohort also had lower hazards of nausea/vomiting (57/695 [8.2%] vs. 140/796 [17.6%]; RR, 0.466; 95% CI, 0.349–0.624; HR, 0.548; 95% CI, 0.402–0.746; *p* < 0.001), antiemetic use (66/251 [26.3%] vs. 129/325 [39.7%]; RR, 0.662; 95% CI, 0.518–0.848; HR, 0.695; 95% CI, 0.516–0.937; *p* = 0.016), gastroesophageal reflux disease (62/556 [11.2%] vs. 150/652 [23.0%]; RR, 0.485; 95% CI, 0.369–0.637; HR, 0.557; 95% CI, 0.413–0.750; *p* < 0.001), constipation (61/969 [6.3%] vs. 126/1002 [12.6%]; RR, 0.501; 95% CI, 0.374–0.671; HR, 0.639; 95% CI, 0.469–0.871; *p* = 0.004), diarrhea (51/861 [5.9%] vs. 118/972 [12.1%]; RR, 0.488; 95% CI, 0.356–0.669; HR, 0.591; 95% CI, 0.425–0.824; *p* = 0.002), bowel obstruction/ileus (15/1303 [1.2%] vs. 50/1296 [3.9%]; RR, 0.298; 95% CI, 0.168–0.529; HR, 0.374; 95% CI, 0.209–0.668; *p* = 0.001), and upper gastrointestinal bleeding (20/1212 [1.7%] vs. 66/1273 [5.2%]; RR, 0.318; 95% CI, 0.194–0.522; HR, 0.404; 95% CI, 0.244–0.668; *p* < 0.001). Dyspepsia/functional dyspepsia (24/1236 [1.9%] vs. 35/1297 [2.7%]; RR, 0.720; 95% CI, 0.431–1.203; HR, 0.956; 95% CI, 0.565–1.619; *p* = 0.868) and irritable bowel syndrome (23/1188 [1.9%] vs. 38/1268 [3.0%]; RR, 0.646; 95% CI, 0.387–1.078; HR, 0.851; 95% CI, 0.503–1.439; *p* = 0.547) did not differ significantly on survival analysis.

### 3.4. Hepatobiliary, Metabolic, Nutritional, Cardiovascular, and Renal Outcomes

Cholelithiasis (26/1108 [2.3%] vs. 59/1048 [5.6%]; RR, 0.417; 95% CI, 0.265–0.656; HR, 0.503; 95% CI, 0.316–0.801; *p* = 0.003), cholecystitis (13/1294 [1.0%] vs. 34/1316 [2.6%]; RR, 0.389; 95% CI, 0.206–0.733; HR, 0.455; 95% CI, 0.239–0.865; *p* = 0.014), and cholecystectomy (28/1251 [2.2%] vs. 81/1265 [6.4%]; RR, 0.350; 95% CI, 0.229–0.533; HR, 0.374; 95% CI, 0.243–0.576; *p* < 0.001) were less frequent in the GLP-1 receptor agonist cohort. Insulin initiation (31/1178 [2.6%] vs. 113/1210 [9.3%]; RR, 0.282; 95% CI, 0.191–0.416; HR, 0.322; 95% CI, 0.216–0.480; *p* < 0.001) and hypoglycemia (12/1343 [0.9%] vs. 38/1366 [2.8%]; RR, 0.321; 95% CI, 0.169–0.612; HR, 0.438; 95% CI, 0.227–0.846; *p* = 0.012) were also lower, whereas no events of diabetic ketoacidosis/hyperosmolar hyperglycemic state occurred in either group. Malnutrition (13/1313 [1.0%] vs. 105/1235 [8.5%]; RR, 0.116; 95% CI, 0.066–0.206; HR, 0.136; 95% CI, 0.076–0.242; *p* < 0.001), abnormal weight loss (22/1285 [1.7%] vs. 64/1230 [5.2%]; RR, 0.329; 95% CI, 0.204–0.531; HR, 0.380; 95% CI, 0.233–0.618; *p* < 0.001), and anemia (48/880 [5.5%] vs. 136/893 [15.2%]; RR, 0.358; 95% CI, 0.261–0.491; HR, 0.431; 95% CI, 0.310–0.601; *p* < 0.001) were lower, whereas vitamin D deficiency showed no difference in risk analysis (75/859 [8.7%] vs. 79/1034 [7.6%]; RR, 1.143; 95% CI, 0.844–1.547; *p* = 0.387) but a higher hazard on survival analysis (HR, 1.556; 95% CI, 1.129–2.145; *p* = 0.007). Osteoporosis was lower in risk analysis (20/1300 [1.5%] vs. 38/1280 [3.0%]; RR, 0.518; 95% CI, 0.303–0.886; *p* = 0.014) but not in survival analysis (HR, 0.738; 95% CI, 0.427–1.275; *p* = 0.275). Heart failure (24/1269 [1.9%] vs. 55/1279 [4.3%]; RR, 0.440; 95% CI, 0.274–0.706; HR, 0.587; 95% CI, 0.361–0.953; *p* = 0.029) and acute kidney injury (25/1194 [2.1%] vs. 80/1153 [6.9%]; RR, 0.302; 95% CI, 0.194–0.469; HR, 0.402; 95% CI, 0.255–0.633; *p* < 0.001) were lower in the GLP-1 receptor agonist cohort. Chronic kidney disease (27/1255 [2.2%] vs. 46/1243 [3.7%]; RR, 0.581; 95% CI, 0.364–0.929; *p* = 0.022; HR, 0.754; 95% CI, 0.466–1.218; *p* = 0.247), myocardial infarction (10/1356 [0.7%] vs. 23/1356 [1.7%]; RR, 0.435; 95% CI, 0.208–0.910; *p* = 0.023; HR, 0.591; 95% CI, 0.279–1.252; *p* = 0.165), and stroke (12/1353 [0.9%] vs. 20/1364 [1.5%]; RR, 0.605; 95% CI, 0.297–1.232; *p* = 0.162; HR, 0.903; 95% CI, 0.435–1.876; *p* = 0.784) were not significantly different on survival analysis.

## 4. Discussion

In this study of non-diabetic adults with chronic pancreatitis, GLP-1 receptor agonist exposure was associated with lower hazards of recurrent acute pancreatitis, pancreatic cancer, all-cause mortality, emergency room visits, hospitalization, ERCP, chronic opioid prescriptions, insulin initiation, hypoglycemia, malnutrition, abnormal weight loss, anemia, heart failure, and acute kidney injury. Several gastrointestinal and hepatobiliary outcomes were also less frequent, whereas dyspepsia, irritable bowel syndrome, osteoporosis, chronic kidney disease, myocardial infarction, and stroke were not significantly different on time-to-event analysis. Vitamin D deficiency was the only outcome with a higher hazard in the exposed cohort. This findings should be interpreted as a description of recorded outcomes after GLP-1 RA exposure among selected non-diabetic patients with CP, not as evidence of a CP-directed therapeutic effect. The indication for GLP-1 RA therapy in this non-diabetic cohort requires careful interpretation. Because patients with type 1 or type 2 diabetes were excluded, GLP-1 RA exposure should not be interpreted as diabetes treatment or as chronic pancreatitis-directed therapy. The higher baseline frequency of obesity-related diagnoses, BMI-coded categories, obstructive sleep apnea, dyslipidemia, hypertension, and other cardiometabolic conditions in the exposed cohort suggests that GLP-1 RA prescribing likely reflected weight-management or cardiometabolic indications. These may benefit CP as well due to the fact that obesity and dyslipidemia are modifiable risk factors for CP, as was likely the case in our study. However, TriNetX does not provide a prescribing indication, and the absence of a recorded obesity diagnosis does not exclude overweight, uncoded obesity, weight-related comorbidity, or prescriptions recorded without a linked indication. Therefore, indication-related selection bias and residual confounding remain important limitations despite post-match covariate balance.

The pancreatic, oncologic, and mortality findings are notable but require careful interpretation. Chronic pancreatitis is a high-burden disease in which pain-related disability, repeated healthcare use, and long-term pancreatic cancer risk are central concerns [2,4]. The present data do not show an excess of recorded recurrent acute pancreatitis or pancreatic cancer; however, these findings should not be interpreted as direct evidence that GLP-1 receptor agonists prevent pancreatitis recurrence or cancer. Future Prospective Validation for these hypotheses are required. The follow-up duration may be insufficient for long-latency pancreatic or gastrointestinal cancer outcomes, and TriNetX does not capture surveillance intensity, imaging frequency, endoscopy frequency, pancreatic morphology, or reasons for diagnostic workup. Differences in surveillance, detection, competing risks, residual confounding, and survivor bias may therefore have contributed to the observed cancer and mortality associations to some extent. Earlier pharmacovigilance and administrative database analyses raised concern regarding pancreatitis or pancreatic malignancy with incretin-based therapies [7,8], whereas more recent population-based studies have not shown a clear increase in acute pancreatitis or pancreatic cancer with incretin-based therapy [9,11,12]. Despite the fact that GLP1 RA has an overall benefit on metabolic outcome and hence by proxy on CP, the size of the mortality association and the simultaneous reductions in pancreatitis, cancer, healthcare utilization, etc., are unlikely to be explained by a single direct drug mechanism in this retrospective design. These estimates may instead reflect some degree of residual confounding by disease severity, prescribing selection, differential healthcare contact, detection bias, competing risks, or survivor bias.

The reductions in emergency care, hospitalization, ERCP, and chronic opioid prescriptions are potentially clinically relevant. Pain phenotype and pain-associated complications are major drivers of disability and resource utilization in chronic pancreatitis [2], and the lower hazards observed here suggest that GLP-1 receptor agonist exposure may identify a subgroup with a more favorable clinical course. Similarly, the lower risks of malnutrition, abnormal weight loss, and anemia are relevant because pancreatic exocrine insufficiency and nutritional failure are linked to adverse outcomes and increased mortality in chronic pancreatitis [3]. However, malnutrition is visit- and coding-pattern-sensitive. These signals are hypothesis-generating, and should not be judged as direct treatment effects because unmeasured differences in disease severity, obesity phenotype, healthcare engagement, and prescribing behavior may still remain after matching.

In this cohort, GLP-1 receptor agonist exposure was associated with lower hazards of nausea/vomiting, GERD, constipation, diarrhea, bowel obstruction/ileus, and upper gastrointestinal bleeding, whereas randomized obesity studies and pooled safety analyses have generally identified gastrointestinal adverse events as the most common treatment-emergent effects of semaglutide and tirzepatide [5,6,13]. This apparent divergence likely reflects important differences between trial populations and the present cohort, including background pancreatic disease, outcome definitions based on coded clinical encounters, and residual confounding by indication, rather than evidence that GLP-1 receptor agonists broadly improve all upper and lower gastrointestinal symptoms in chronic pancreatitis. The isolated higher hazard for vitamin D deficiency should likewise be viewed cautiously, particularly because the corresponding risk analysis was not statistically significant and follow-up was shorter in the exposed cohort.

The direction of the cardiovascular and renal findings is broadly consistent with the wider GLP-1 receptor agonist literature. Studies have shown cardiovascular benefit with semaglutide in obesity without diabetes, symptomatic improvement in obesity-related HFpEF, and kidney benefit in type 2 diabetes with chronic kidney disease [14,15,16]. In our study, heart failure and acute kidney injury were significantly lower in the GLP-1 receptor agonist cohort, whereas chronic kidney disease, myocardial infarction, and stroke were not significantly different on survival analysis.

The CP-specific literature is limited and does not support a definitive clinical indication yet. A recent TriNetX study of 89,596 patients with CP reported a lower 5-year pancreatic cancer incidence among GLP-1 RA users after propensity score matching (HR 0.49, 95% CI 0.30–0.80), including in the subgroup with CP and type 2 diabetes (HR 0.53, 95% CI 0.31–0.91), but the authors emphasized the need for prospective confirmation, as do we based on the results of our study [17]. Mechanistic data are also not uniformly reassuring: Nakamura et al. showed increased GLP-1 receptor expression in acute and chronic pancreatitis tissue and in activated pancreatic stellate cells; liraglutide did not induce inflammatory gene expression in activated stellate cells, but it did induce stellate-cell proliferation, making biological interpretation uncertain rather than clearly protective [18]. In patients with prior acute pancreatitis, recent studies have reported lower recurrent acute pancreatitis after semaglutide or tirzepatide and suggested that GLP-1 RAs may be used in selected patients with previous acute pancreatitis [19,20]. However, those studies were not designed specifically for established non-diabetic CP. Conversely, a large federated-network study in obesity or type 2 diabetes reported lower all-cause mortality but a small increased acute pancreatitis risk, no significant difference in chronic pancreatitis or pancreatic cancer after matching, and early time-varying risk patterns [21]. Another study reported modestly increased acute pancreatitis and pancreatic cancer risk after GLP-1 RA initiation compared with DPP-4 inhibitors and specifically recommended vigilance when GLP-1 RAs are used for off-label indications [22]. Taken these together, the current literature is mixed, largely observational, and does not establish GLP-1 RAs as CP-directed therapy; our data should therefore be viewed as an additional real-world signal rather than confirmation of safety or benefit. However, treatment selection, healthy-user and selective-prescribing effects, unmeasured disease severity, and differences in healthcare engagement persist and may have also influenced the analysis [23,24].

This study has several strengths, including a large multicenter real-world network, exclusion of patients with diabetes, broad outcome capture, and post-match covariate balance with standardized mean differences below 0.1. However, the limitations are substantial. First, the retrospective design precludes causal inference. Second, the GLP-1 receptor agonist and comparator cohorts differed markedly before matching, especially in obesity-related and cardiometabolic characteristics, indicating major confounding by indication and selection bias. Propensity score matching balanced measured covariates but cannot address unmeasured factors such as exact prescribing indication, BMI severity beyond available codes, weight trajectory, chronic pancreatitis severity, imaging phenotype, pain burden, nutritional status, alcohol exposure, smoking intensity, socioeconomic status, healthcare engagement, medication dose, duration, and adherence. Third, the index-date definitions were not symmetric between cohorts, and mean follow-up was shorter in the GLP-1 RA cohort (458.8 vs. 664.4 days after matching). Therefore, time-zero bias, immortal-time bias, survivor bias, and differential observation time cannot be excluded. Outcome-specific exclusion of prevalent events also led to varying at-risk denominators across analyses. Fourth, diagnosis, procedure, medication, and mortality coding may misclassify exposure or outcomes, and TriNetX may incompletely capture care received outside participating organizations. Fifth, the cancer and mortality findings should be considered exploratory. The cancer follow-up period may be insufficient for long-latency outcomes, and the magnitude of the mortality association is unlikely to represent a true causal treatment effect in this observational database study. Sixth, although emerging studies have examined GLP-1 RA exposure in patients with CP or prior acute pancreatitis, the literature is sparse, mostly retrospective, and not specific to non-diabetic CP across the broad outcome range evaluated here. The available data do not establish GLP-1 RAs as safe, protective, or therapeutically indicated in CP. Differential follow-up, detection bias, differential surveillance, healthy-user bias, survivor bias, incomplete mortality capture, and unmeasured differences in baseline frailty or disease severity may have contributed to the observed estimates. Although E-value analyses quantify the degree of unmeasured confounding needed to explain selected associations, they do not address these other sources of bias. Because the TriNetX database did not permit validation of indication, dose, duration, adherence, time-varying exposure, surveillance intensity, or CP severity, these concerns remain.

In non-diabetic adults with CP, recorded GLP-1 RA exposure was associated with lower hazards of several coded pancreatic, oncologic, healthcare utilization, nutritional, gastrointestinal, cardiovascular, and renal outcomes. However, the literature on GLP-1 RA use in CP remains limited and inconsistent, and the present study cannot establish safety, pancreatic protection, or a therapeutic indication. Prospective studies with defined indication are required before any clinical inference can be made.

## 5. Conclusions

Among non-diabetic adults carrying a recorded chronic pancreatitis diagnosis, those with recorded GLP-1 RA exposure had lower recorded hazards across many clinically relevant outcomes. However, this is an observation of clinical coding of the outcomes. The breadth and magnitude of the associations, small event counts for key endpoints, shorter follow-up, non-adjudicated disease definition, and susceptibility to selection, healthy-user, time-zero, survivor, surveillance, and coding biases preclude causal interpretation. These findings do not establish pancreatic safety, protection, or a therapeutic indication for GLP-1 RAs in chronic pancreatitis and should be considered hypothesis-generating only. Future prospective studies are required to confirm these hypotheses.

## Figures and Tables

**Table 1:** Baseline characteristics before and after propensity score matching.

Characteristic	Before PSM: GLP-1 RA (*N* = 1441)	Before PSM: Non–GLP-1 RA (*N* = 142,047)	SMD	After PSM: GLP-1 RA (*N* = 1422)	After PSM: Non–GLP-1 RA (*N* = 1422)	SMD
Age, years (mean ± SD)	54.0 ± 13.3	54.2 ± 17.7	0.012	54.0 ± 13.3	55.3 ± 15.4	0.091
Female, *n* (%)	1047 (72.7%)	68,201 (50.1%)	0.476	1031 (72.5%)	1040 (73.1%)	0.014
Male, *n* (%)	393 (27.3%)	67,852 (49.9%)	0.477	390 (27.4%)	382 (26.9%)	0.013
White, *n* (%)	1126 (78.1%)	98,300 (72.2%)	0.137	1109 (78.0%)	1112 (78.2%)	0.005
Black, *n* (%)	186 (12.9%)	20,209 (14.8%)	0.056	185 (13.0%)	178 (12.5%)	0.015
Hypertension, *n* (%)	842 (58.4%)	40,747 (29.9%)	0.599	828 (58.2%)	867 (61.0%)	0.056
Dyslipidemia, *n* (%)	790 (54.8%)	27,564 (20.3%)	0.764	773 (54.4%)	798 (56.1%)	0.035
Obesity (E66.0), *n* (%)	649 (45.0%)	3529 (2.6%)	1.149	631 (44.4%)	662 (46.6%)	0.044
BMI 30–39, *n* (%)	583 (40.5%)	5570 (4.1%)	0.972	566 (39.8%)	585 (41.1%)	0.027
BMI ≥ 40, *n* (%)	330 (22.9%)	1754 (1.3%)	0.703	317 (22.3%)	312 (21.9%)	0.008
OSA, *n* (%)	460 (31.9%)	5170 (3.8%)	0.789	444 (31.2%)	449 (31.6%)	0.008
CHD, *n* (%)	253 (17.6%)	11,020 (8.1%)	0.286	247 (17.4%)	238 (16.7%)	0.017
Heart failure, *n* (%)	149 (10.3%)	6168 (4.5%)	0.223	146 (10.3%)	130 (9.1%)	0.038
CKD, *n* (%)	165 (11.5%)	7506 (5.5%)	0.214	164 (11.5%)	165 (11.6%)	0.002
COPD, *n* (%)	162 (11.2%)	9433 (6.9%)	0.150	158 (11.1%)	146 (10.3%)	0.027
Alcohol-related disorders, *n* (%)	203 (14.1%)	20,443 (15.0%)	0.026	202 (14.2%)	205 (14.4%)	0.006
Tobacco use, *n* (%)	124 (8.6%)	7164 (5.3%)	0.132	122 (8.6%)	109 (7.7%)	0.033
Cholelithiasis, *n* (%)	305 (21.2%)	10,165 (7.5%)	0.399	299 (21.0%)	326 (22.9%)	0.046
EPI, *n* (%)	91 (6.3%)	967 (0.7%)	0.308	77 (5.4%)	79 (5.6%)	0.006

**Table 2:** Summary of all outcomes.

Outcome	HR (95% CI)	*p*-Value
**Pancreatic Outcomes**		
Recurrent acute pancreatitis	0.247 (0.149, 0.409)	<0.001
Pancreatic cancer	0.255 (0.128, 0.511)	<0.001
**Mortality**		
All-cause mortality	0.167 (0.105, 0.263)	<0.001
**Healthcare Utilization**		
ER visits	0.441 (0.311, 0.626)	<0.001
Hospitalization	0.428 (0.298, 0.615)	<0.001
ERCP	0.166 (0.088, 0.313)	<0.001
**Opioid-Related**		
Chronic opioid prescriptions	0.522 (0.338, 0.804)	0.003
Opioid use disorder	0.437 (0.212, 0.898)	0.021
**GI Symptoms**		
Nausea/Vomiting	0.548 (0.402, 0.746)	<0.001
Antiemetic use	0.695 (0.516, 0.937)	0.016
GERD	0.557 (0.413, 0.750)	<0.001
Dyspepsia	0.956 (0.565, 1.619)	0.868
Constipation	0.639 (0.469, 0.871)	0.004
Diarrhea	0.591 (0.425, 0.824)	0.002
IBS	0.851 (0.503, 1.439)	0.547
Bowel obstruction/ileus	0.374 (0.209, 0.668)	0.001
Upper GI bleed	0.404 (0.244, 0.668)	<0.001
**Hepatobiliary**		
Cholelithiasis	0.503 (0.316, 0.801)	0.003
Cholecystitis	0.455 (0.239, 0.865)	0.014
Cholecystectomy	0.374 (0.243, 0.576)	<0.001
**Metabolic/Endocrine**		
Insulin initiation	0.322 (0.216, 0.480)	<0.001
Hypoglycemia	0.438 (0.227, 0.846)	0.012
DKA/HHS	N/A (0 events)	1.000
**Nutritional/Hematologic**		
Malnutrition	0.136 (0.076, 0.242)	<0.001
Abnormal weight loss	0.380 (0.233, 0.618)	<0.001
Vitamin D deficiency	1.556 (1.129, 2.145)	0.007
Anemia	0.431 (0.310, 0.601)	<0.001
Osteoporosis	0.738 (0.427, 1.275)	0.275
**Cardiovascular/Renal**		
Heart failure	0.587 (0.361, 0.953)	0.029
AKI	0.402 (0.255, 0.633)	<0.001
CKD	0.754 (0.466, 1.218)	0.247
MI	0.591 (0.279, 1.252)	0.165
Stroke	0.903 (0.435, 1.876)	0.784

## Data Availability

The data presented in this study are available in TriNetX platform at https://trinetx.com/.
